# Virtual Training on Stress Management for the Mothers of Children with Disabilities in the United Arab Emirates

**DOI:** 10.3390/ijerph20021450

**Published:** 2023-01-13

**Authors:** Rawhi Abdat, Maxwell Peprah Opoku, Mohammed Safi, Siham Al Harballeh, Rhoda Myra Garces-Bacsal

**Affiliations:** 1 Rehabilitation of Persons with Disabilities Department, Ministry of Community Development Dubai, Dubai P.O. Box 17781, United Arab Emirates; 2 Department of Special Education, College of Education, United Arab Emirates University, Al Ain P.O. Box 15551, United Arab Emirates; 3 Department of Speech Language Pathology, United Arab Emirates University, Al Ain P.O. Box 15551, United Arab Emirates

**Keywords:** stress management, parents, children with disability, intervention, United Arab Emirates

## Abstract

Parenting children with disabilities has consistently been reported to be associated with stress, and even more so among mothers who are primary caregivers. In non-Western contexts such as the United Arab Emirates (UAE), there is a dearth of research on maternal mental health in relation to raising children with disabilities and related mitigation strategies. During the COVID-19 pandemic (2020–2021), the Federal Government of the UAE used the Save the Child’s Stress Checklist to assess the stress levels of 635 mothers who were raising children with disabilities in the northern part of the country. In the pre-test, an estimated 6% (36/635) of the mothers were found to be experiencing high levels of stress. Consequently, virtual stress management training was conducted, and 12 of the 36 mothers completed the full training. Wilcoxon’s ranked test showed a reduction in their total, physical, emotional and cognitive stress at the end of the training. This study demonstrates the pressing need for ongoing training programmes to improve the mental health of mothers of children with disabilities in the UAE and similar contexts.

## 1. Introduction

Stress refers to the body’s response to harmful stimuli, and such responses can appear in the form of physical, social, psychological or cultural reactions [1,2,3]. According to Linden [1], triggers from both internal and external forces contribute to these reactions in individuals. The body’s reaction to stress can lead to the malfunctioning of some body parts and even disease. Numerous factors contribute to stress, such as witnessing violence, disasters, crime, exposure to trauma and profound difficulties, including giving birth to a child with a disability [2]. Due to the adverse effects of stress on health and even performance [3,4,5,6,7], suggestions have been made regarding the need for continuous monitoring and support to enhance the wellbeing of individuals in society [3].

The corpus of literature has reported high stress levels among parents raising children with disabilities [4,8,9,10,11,12,13,14]. For example, the birth of a child with a disability means that at least one parent will be restricted to providing full-time care on a day-to-day basis [15,16,17,18], which could then affect the parents’ social and professional lives [17,18]. In some families, tensions can arise between the parents raising children with disabilities, as they may struggle to provide care for these children [15], which could have repercussions for family unity and cohesion [15,19]. Moreover, in some societies, the birth of a child with a disability is linked to superstitions or spirituality [17,20]. Such perceptions have a negative impact on parents who have to contend with raising children with disabilities as well as negative public perceptions. Furthermore, evidence suggests that parents do not have the requisite training to support the development of their children [15,16,17,18,19,20]. Most importantly, some parents lack an understanding of the disability as well as the ways to provide their children with independent living skills [18]. In some situations, parents do not have enough information about the avenues they can use to access services for their children with disabilities [21]. This lack of capacity among parents to obtain help and support in raising their children with disabilities has been described as a major source of stress for these parents.

The outbreak of COVID-19 heightened the discussion on parental stress associated with raising children with disabilities [5,6,7,22,23,24,25,26,27]. The subsequent isolation brought about by the pandemic compounded the stress levels of parents with children with disabilities [27,28,29,30,31,32]. For instance, Dhiman et al. [7] aimed to investigate the impact of the COVID-19 pandemic on the mental health of caregivers of children with disabilities. In addition to other symptoms connected to anxiety and stress, they found high depressive symptoms among caregivers. Kim et al. [26] conducted in-depth interviews with the parents of children with intellectual disabilities to understand their concerns regarding the limited services provided to their children during the COVID-19 restrictions. The parents expressed their concerns about the challenges they faced with their children, such as the loss of independent living skills, the lack of communication support, the dearth of opportunities for physical activities, increasing behavioural problems and isolation from the larger society. In other words, the outbreak of COVID-19 seemed to compound the stress levels of these parents. This underscores the urgent need for formal support systems that consider innovative approaches towards reducing or alleviating stress among such parents in countries [28] such as the United Arab Emirates (UAE). While several studies have been conducted to demonstrate the difficulties faced by parents who are raising children with disabilities and the contribution of such circumstances to an increase in their stress levels in the UAE [19,29], there is little evidence on stress mitigation strategies.

The UAE government pays considerable attention to the needs and wellbeing of parents of children with disabilities. For example, the National Policy for Empowering People of Determination (a term that is used to refer to people with disabilities in the UAE) contains an entire pillar of social protection and family empowerment which includes social security policies and family engagement in rehabilitative services through the organisation of support programs, training and consultation [30]. Moreover, the Advisory Council for the People of Determination in the UAE enables people of determination and their families to participate in the development and implementation of national policies and initiatives for people of determination in the country. The representation of the parents of children with disabilities on this platform allows them to elucidate the challenges they are facing and to offer useful guidelines to address their challenges. In addition, the quality standards of early childhood interventions in the UAE ensure the continuous development and empowerment of the parents of children with disabilities to equip them with the skills required to support the development of their children [31].

During the COVID-19 pandemic, the government closed schools and services used for the rehabilitation of children with disabilities. In the post COVID-19 era, children with disabilities take some classes from home and attend school on other days. This school arrangement and inherent challenges associated with raising children with disabilities could place additional burdens on the primary caregivers of children with disabilities. Consequently, the UAE government recognised the importance of the need for a stress management intervention system and developed a response policy for people with disabilities and their families in emergencies, crises and disasters [32]. The policy offers an educational awareness platform for the parents of children with disabilities to equip them with specialised information on the mechanisms for dealing with their children in crisis situations as well as stress alleviation strategies [32,33]. It is against this backdrop that this experimental study explored the effectiveness of the stress management training offered to primary caregivers, that is, mothers raising children with disabilities in the UAE.

The post-COVID-19 era has led to the integration of virtual platforms such as learning management systems, Microsoft teams, Zoom, social media and others in all spheres of life, particularly in training and development [34,35]. With most mothers serving as the primary carers of children in the UAE [19], the Federal Government of the UAE through the Ministry of Community Development (MCD) deemed it appropriate to offer stress management training virtually in order to reach a large population. However, the stress levels of participants were recorded before and after the training in order to provide baseline information about the effectiveness of the stress management intervention. The study was guided by the following research questions:

1. What are the stress levels of mothers raising children with disabilities in the UAE?
2. What is the association between background variables and the stress levels of mothers raising children with disabilities in the UAE?
3. Will the stress intervention training impact the stress levels of mothers raising children with disabilities in the UAE?

## 2. Materials and Methods

### 2.1. Study Participants

The study participants comprised mothers who were raising children with disabilities in the UAE. The government has a database of parents who are raising children with disabilities. The study targeted parents who had enrolled their children with disabilities in public special schools/rehabilitation facilities across the country. The decision to target this population was due to changes in the school system and the possibility of parents needing to make life adjustments in an effort to continue nurturing their children with disabilities. Since mothers are mainly the primary caregivers in the UAE context, mothers who were raising children with disabilities below the age of 18 were the targets of this study. During the 2020/2021 academic year, 814 mothers met the inclusion criteria, but only 635 mothers of children with disabilities consented to participate in the first phase of the study.

### 2.2. Study Design and Instrument

**Figure 1:**
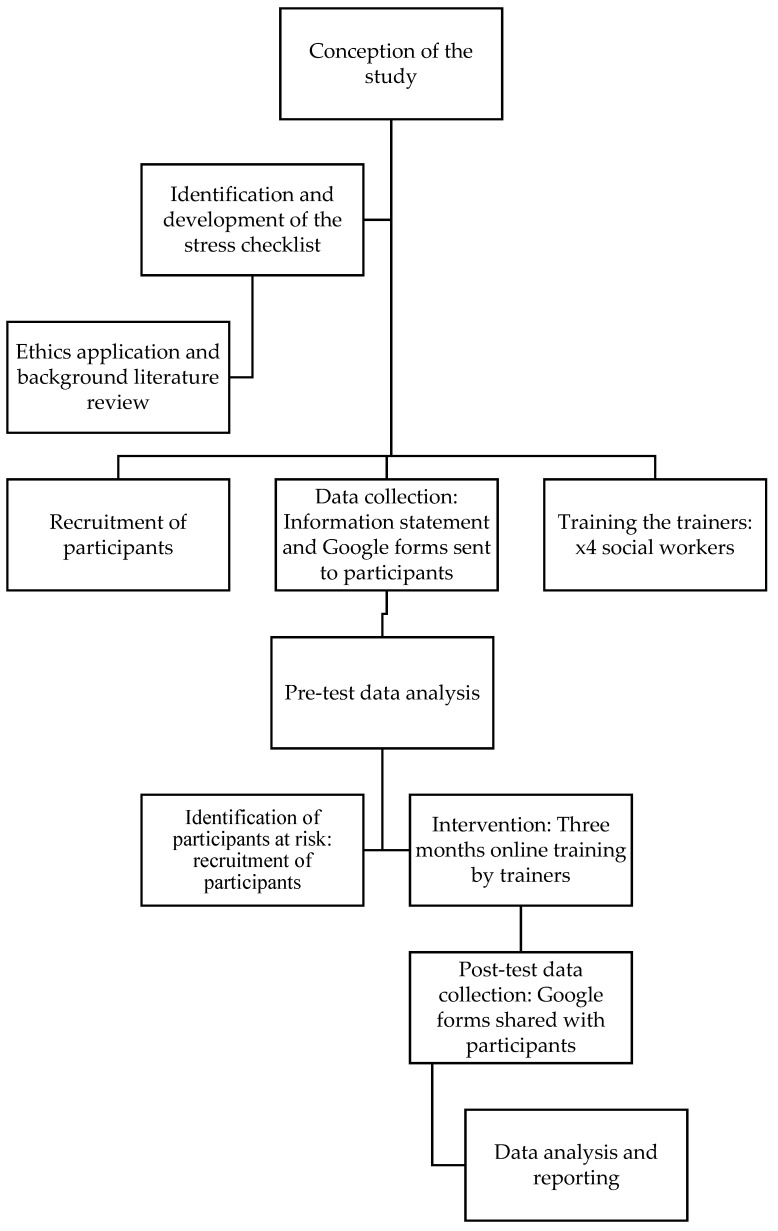
Flow chart of the study design.

A quasi-experimental design was used to investigate whether the training intervention would impact the stress levels of mothers raising children with disabilities in the UAE (see Figure 1). This study was necessary because the government of the UAE has invested resources to support the wellbeing of parents and their children with disabilities. Since the parents of children with disabilities are at risk of stress, the study provided an opportunity to assess whether the training programme institutionalised by the government of the UAE had been effective in decreasing the stress levels of these parents.

Data were collected in two phases using the Save the Child’s Stress Checklist, which was developed by Terlonge and Jørgensen [3]. This scale includes 15 items which represent the experiences of mothers and is anchored on a three-point Likert scale: never [1], sometimes [2] and often [3]. The checklist has three subscales: emotional stress, physical stress and cognitive stress (see Table 1). The instrument was completed pre-training and post-training.

The sum of the scores ranges from 15 to 45, where a higher score indicates a higher stress level: a score of under 20 indicates no signs of stress, 20–35 signifies mild/moderate signs of stress and 36–45 indicates severe stress levels. The actual mean was also calculated to develop additional insight into the mothers’ stress levels. This was achieved by dividing the sum mean by the number of items.

Content validity was used to validate the scale. This was achieved through a review of the scale by three experts to determine the appropriateness of the scale for this study. The feedback from the experts was incorporated into the final draft that was used for data collection.

### 2.3. Training Intervention

The first author provided a one-day training course to social workers at selected rehabilitation centres on the Etma’nno (an Arabic phrase meaning ‘Don’t worry, everything will be fine’) guidelines. Based on these guidelines, the social workers started organising virtual counselling sessions for the targeted mothers. The stress-relief training programme is founded on the Etma’nno guidelines developed by the Ministry of Community Development (MOCD) [36] as a support programme targeting the parents of children with disabilities. The MOCD published these guidelines to equip the parents of children with disabilities with coping strategies and techniques to alleviate stress [36]. Using the guidelines, mothers are able to share their emotions and feelings constructively to relieve stress and are empowered with knowledge of stressors, strategies and exercises that can help them overcome their stress.

There are five parts to the programme. Part one covers how parents could understand their children’s behaviour in times of crisis. Parents are able to learn about the sources of anxiety that can lead to crises as well as ways to observe the behaviour of their children to identify the extent of their need for help.

Part two provides guidelines to mothers on ‘do’s and don’ts’ in order to support their children with disabilities. Parents are given a list of words to use and those not to use in difficult times.

Part three focuses on methods of coping with crisis. It contains a set of techniques that mothers can use, such as psychodrama, painting, songs, the retrieval of experiences and imagination.

Part four encompasses the use of social stories, which are essentially illustrated stories that help children understand the crisis stage and coexist with daily life situations through narratives.

Part five focuses on home activities and their therapeutic benefits. This section presents a set of activities that family members can carry out at home to release negative emotions and delineates how to re-channel children’s energy to more purposeful and productive activities [36].

After the pre-test, mothers who were found to be experiencing high levels of stress [33,34,35,36,37,38,39,40,41,42] in the pre-test were considered for participation in the training. During the training, each mother received 24 relief sessions over the course of three months, which comprised two sessions per week, with each session lasting between 30 and 45 min. These virtual sessions were conducted by four professional social workers, each with more than ten years’ experience.

### 2.4. Procedure

The study and its protocols were approved by the university’s Social Science Ethics Review Committee. The MOCD sent a formal email to the parents to inform them about the objectives of the study and its relevance to the UAE community and to encourage their participation. Google Forms were subsequently sent to all the parents for completion by mothers who were the primary caregivers of children with disabilities. The data were collected between February and December 2021. All the participants were informed that taking part in the study was voluntary and that they could withdraw from it at any time without any negative consequences. They were also informed that they would not receive any reimbursement for taking part in this study. They were assured that their identity and personal information would not be shared with anyone outside the research team. It was further explained to them that neither their identity nor personal information would be used in the reporting of this study. In the first phase, each participant was given a code, which was used to trace those experiencing stress who were included in the second phase. The estimated time to complete the instrument was 15 min, and those who were found to be experiencing high levels of stress were informed that they would be invited to participate in stress management training.

### 2.5. Data Analysis

Google Forms was used for data collection, and these data were transferred to Excel to be cleaned and to compute the level of stress of each participant. The data of those who scored within the range of 36–45 were extracted into a different file, and these participants were contacted directly to enrol them in the next stage of the study.

To answer research question 1, the data were transferred to SPSS to calculate the total stress mean score and the subscale scores. Since the sample for the first phase was large, it was deduced that the data were normally distributed [37].

To answer research question 2, a one-way analysis of variance (ANOVA) was undertaken to understand whether the place of residence and the child’s disability type could influence the participants’ stress levels. Homogeneity of variance was assessed to ensure that it was not violated. The relationship between the subscales was also assessed using correlation analysis, and the results were interpreted as follows: small (0.10–0.30), medium (0.31–0.50) and large (at least 0.51) [37].

The participants who were experiencing high levels of stress were enrolled in the stress management training. A month after completing the training, they were invited to complete the stress checklist [37]. To answer research question 3, given the small sample size, the Wilcoxon signed rank test was used to assess the difference between the pre- and post-test results of those who participated in the training [37]. This was carried out to check whether the training impacted the stress levels of the participants.

## 3. Results

The reliability of the scale was calculated using Cronbach alpha, which yielded the following scores: total stress = 0.92, emotional stress = 0.83, physical stress = 0.70 and cognitive stress = 0.81.

The results further showed a very large correlation between the subscales: emotional stress and physical stress (r = 0.77, *p* = 0.001), emotional stress and cognitive stress (r = 0.76, *p* = 0.001) and physical stress and cognitive stress (r = 0.73, *p* = 0.001).

### 3.1. Pre-Test

The frequency counts indicated that 31% (200) of the parents reported having no signs of stress, namely they obtained a score of 20 or below. On the other hand, while 63% (399; stress between 21 and 30) reported mild/moderate stress, 6% (36; score between 36 and 45) of the participants were found to have high stress levels.

With regard to the subscales, the results for emotionality were as follows: 16% (105) of the participants had no stress, 67% (424) were experiencing mild/moderate stress and 17% (106) felt high levels of stress. For physical stress, 16% (103) of the participants had no stress, 69% (437) were experiencing mild/moderate stress and 15% (95) of the participants felt high levels of stress. With respect to cognitive stress, the results were as follows: 23% (149) of the participants had no stress, 59% (376) had mild/moderate stress and 18% (110) had high stress.

The mean score for the total stress level was 1.63 (SD = 0.45), and the subscales yielded the following scores: emotional stress: M = 1.60, SD = 0.48; physical stress: M = 1.71, SD = 0.61; cognitive stress: M = 1.65, SD = 0.67 (see Table 1).

A one-way between-groups ANOVA was calculated to ascertain whether the participants’ results differed based on their children’s diagnoses. The results showed no differences between the children’s diagnoses (i.e., cognitive, sensory, developmental delay, and multiple disabilities) and the stress levels of mothers (total stress, F (3, 608) = 0.35, *p* = 0.79, partial eta squared = 0.002; emotional stress, F (3, 608) = 0.45, *p* = 0.72, partial eta squared = 0.002; physical stress F (3, 608) = 0.61, *p* = 0.61, partial eta squared = 0.003; and cognitive stress, F (3, 608) = 13, *p* = 0.94, partial eta squared = 0.001).

Furthermore, the ANOVA was calculated to explore the relationship between the place of residence of the participants and their stress levels. While the results showed no differences between the participants in terms of emotional stress (F (5, 629) = 1.92, *p* = 0.09, partial eta squared = 0.02) and physical stress (F (5, 629) = 2.11, *p* = 0.06, partial eta squared = 0.02), differences were found between the participants with respect to total stress (F (5, 629) = 2.34, *p* = 0.04, partial eta squared = 0.02) and cognitive stress (F (5, 629) = 3.07, *p* = 0.01, partial eta squared = 0.02).

A post hoc comparison test was used to determine where the differences may lie between the participants. The total stress of the participants who indicated they were living in Ras Al Khaimah (M = 1.56; SD = 0.40) was different from the total stress of those living in Ajman (M = 1.71; SD = 0.47) only. However, those living in other Emirates (Dubai, M = 1.67, SD = 0.36; Fujairah, M = 1.64, SD = 0.43; Sharjah, M = 1.70, SD = 0.45; Umm Al Quwain, M = 1.71, SD = 0.39) did not differ from one another in terms of total stress.

Similar trends were observed in terms of the differences between the participants for cognitive stress, as those living in Ras Al Khaimah (M = 1.53, SD = 0.52) were different from those living in Ajman (M = 1.77, SD = 0.59). The other Emirates yielded the following results: Dubai (M = 1.61, SD = 0.59), Fujairah (M = 1.67, SD =.55), Sharjah (M = 1.70, SD = 0.56) and Umm Al Quwain (M = 1.71, SD = 0.51).

### 3.2. Post-Test

Stress management training was provided to the mothers who were found to be experiencing high levels of stress. However, out of the 6% (36/635) of the mothers who may have been at risk of stress, only 12 took part in the training and completed the post-test stress rating scale. The frequency counts showed a reduction in the stress scores from high- to mild/moderate-risk levels. Table 2 summarises these post-test results.

The Wilcoxon signed tank test revealed a statistically significant reduction in stress following participation in the training programme: z = −3.11, n = 12, *p* = 0.002, with a very large effect size of 0.90. The median score for stress decreased from before the programme (Md = 39) to after the programme (Md = 29).

Wilcoxon ranked tests were conducted for each of the subscales. The cognitive stress results were as follows: z = −3.13, n = 12, *p* = 0.002, with a very large effect size of 0.90. The median of cognitive stress decreased from the pre-test (Md = 10) to the post-test (Md = 7). With respect to emotional stress, the results were as follows: z = −3.11, n = 12, *p* = 0.002, with a large effect size of 0.89. Emotional stress decreased from the pre-test (Md = 10.50) to the post-test (Md = 16). In terms of physical stress, the output showed z = −2.72, n = 12, *p* = 0.006, with a large effect size of 0.79. Lastly, physical stress decreased from the pre-test (Md = 13) to the post-test (Md = 12).

## 4. Discussion

In this study, insights were gained into the influence of stress management training on the stress levels of the primary caregivers of children with disabilities in the UAE. It is useful to state here that there is an intricate relationship between parenting children with disabilities and stress levels [4,10,11,12,13,14,15]. The stressors have been found to be enormous, including but not limited to finances [17], tension within the family [15], behavioural problems [19] and the isolation of the parents from society [19]. This study confirmed such assertions, with an estimated 63% and 6% of the participants experiencing a mild/moderate and high level of stress, respectively. It is possible that mothers who are raising children with disabilities may be facing difficulties doing so. Indeed, the few studies conducted in the UAE context have reported parental challenges [19,29], which is similar to reports from previous studies conducted in other countries [21,38]. The findings underscore the need for policymakers to engage with parents to understand some of their concerns, as well as to explore possible ways to support their development.

The findings of the study support the relevance of stress management training for parents who are raising children with disabilities. The participants of this study reported reduced stress levels following the training or similar. This finding is consistent with that of another study which reported the relevance of stress management training in reducing or alleviating stress [28]. The stress levels of the participants were also reduced for each of the subscales. This could be linked to the quality of the stress management training that is being implemented in the UAE. As previously explained, the Etma’nno stress relief guidelines [36] encompass five components, which range from understanding children’s behaviour to how parents support the development of their children at home. The nature of the training enables participants to develop insights into the behaviour of their children and to acquire useful support strategies. However, there is room for further improvement as the stress levels of the participants in this study moved from high to mild/moderate levels. Policymakers in the UAE may consider creating a system within which to conduct regular stress management training for the parents of children with disabilities.

Correlations between the subscales were noted in this study. Although the intention is not to suggest causation, the large correlations between the subscales (cognitive, physical and emotional) could provide important insights into the stress levels of the participants. It would be fair to indicate that as cognitive stress rises, physical stress also increases in the same direction and vice versa. A similar conclusion could be drawn in relation to the relationship of the other subscales. According to the literature, multiple factors could explain or contribute to stress among parents who are raising children with disabilities [12,23,26,39]. In view of this, stress can manifest in various ways in individuals such as the participants who took part in this study. It is unsurprising that the developed training, which covers multiple areas, was relevant to the study participants who were found to be at high risk of stress. This finding underscores the need for stakeholders to measure stress levels as well as to use multiple intervention strategies to alleviate stress among mothers who are raising children with disabilities.

The study participants’ place of residence was found to influence their overall and cognitive stress. The participants who indicated that they were living in Ras Al Khaimah reported lower stress levels (and cognitive stress levels) than those living elsewhere. Although it would be difficult to offer a useful explanation for this trend, it does appear that the geographical location of a mother and child can impact their stress levels. Indeed, previous studies have reported the role of geography in the rehabilitation of children with disabilities and their families [40,41]. This finding seems to underscore the need for more contextualised types of support systems for the parents of children with disabilities in the country. Since the UAE is a federal system, each Emirate should pay close attention to the wellbeing of parents who are raising children with disabilities. More targeted approaches could be used to determine the specific stressors that may be unique to a particular Emirate, and this could enable practitioners and policymakers to develop initiatives that are more customised and effective.

One notable finding of this study was that no difference was found between maternal stress levels and the type of disability or diagnosis of the children. This suggests that, regardless of the disability of their child, parents could at any point be at risk of some stress. Previous studies have found that parents who are raising children with disabilities encounter challenges [4,19,20,42]. It is useful to reiterate here that the traditional understanding of disability is dominant in the UAE context [43]. Specifically, the onset of disability is culturally attributed to spiritual or supernatural powers. This has contributed to the subjugation of persons with disabilities and their families in society [43]. It is thus possible that the participants who took part in this study had demonstrated or experienced similar difficulties, which contributed to their stress. This finding lends some support for policymakers to consider all parents who are raising children with disabilities when seeking to enhance their wellbeing.

### Study Limitations

It is challenging to generalise the findings of the study given its limitations. First, the focus of this study was mothers, who are largely the primary caregivers of their children in the UAE context. It was beyond the scope of the current study to include the voices of fathers who are typically the breadwinners in families. Second, the participants only included the mothers of children with disabilities who were enrolled at federal government special rehabilitation centres. The mothers of children with disabilities who were enrolled in private special rehabilitation centres were therefore not included in this study. Third, the study drew on the experiences of participants residing in northern Emirates in the UAE. One of the larger Emirates, Abu Dhabi, which is located in the south, was thus excluded from this study, mainly because it has a separate ministry and support programme for children with disabilities. Nonetheless, future studies may consider expanding the study region to include Abu Dhabi, as well as to incorporate a comparison of the stress levels of both mothers and fathers. Fourth, although the design for this study was quasi-experimental, there was no control group for comparison. A future study could expand this study through the addition of control groups to determine whether the stress training could have impacts on diverse groups.

## 5. Conclusions

Parenting children with disabilities has its own challenges [4,19,20,42], which have been found to increase the stress levels of parents [4,6,26]. This situation was heightened and exacerbated by the recent COVID-19 pandemic. The search for ways to alleviate the burden on parents with children with disabilities has persisted, and the current study demonstrates that mothers may be experiencing psychological stress. A virtual training program was therefore designed based on the Etma’nno first aid psychological guidelines to ascertain its impact on stress alleviation in the UAE context [36]. Significant differences were found between pre- and post-stress-management-training. It could be deduced that the decrease in the stress levels of the participating mothers could be attributed to the intervention programme. The findings of this study could provide valuable information to practitioners regarding the potential implementation of Etma’nno training programmes in schools to support mothers who are raising children with disabilities in the UAE.

Global advocacy has encouraged governments to come up with innovative programmes to support the development of children with disabilities and their families [44,45]. The UAE government has taken an important step which could be encouraged or formalised in the country. The results of the current study could have considerable implications for disability service providers in the UAE. It would be fair to postulate that Etma’nno [36]-based virtual training is a promising method for mitigating stress levels among the mothers of children with disabilities. Policymakers could recommend that the stress checklist used in this study be employed in schools to monitor the stress levels of the parents of children with disabilities. Continuous or ongoing stress management training such as Etma’nno could likewise be adopted by schools to help alleviate the stress levels of parents raising children with disabilities in the UAE. Furthermore, each Emirate could develop a region-specific stress monitoring and training programme for parents and their children with disabilities. There is currently a growing need to develop a more conducive and inclusive environment for children with disabilities. Intervention programmes such as stress management training could enable parents to attain acceptable psychological wellbeing to support and nurture their children with disabilities.

## Figures and Tables

**Table 1:** Summary of the means.

Code	Items	*M*	*SD*
	**Emotional stress**	1.60	0.48
SC_1	I feel anxious or nervous.	1.85	0.58
SC_4	I feel irritable [e.g., little noises make me jumpy].	1.63	0.68
SC_5	I feel sad and want to cry.	1.60	0.66
SC_6	I have lost my sense of humour.	1.50	0.66
SC_8	I feel defeated or afraid, and I yearn for a place where I can feel safe.	1.51	0.66
SC_15	I am quick to feel resentment, and any small problem or request annoys me greatly [e.g., I overreact to other people’s mistakes or notice that I fight with my family more than usual].	1.54	0.65
	**Physical stress**	1.71	0.61
SC_2	I suffer from several physical problems [e.g., headache, rapid palpitations, chest pain, stomach aches].	1.90	0.66
SC_3	I feel tired and have chronic fatigue even after I get enough sleep.	1.75	0.69
SC_9	I act recklessly and take risks where I shouldn’t.	1.29	0.53
SC_12	I am less efficient and organised at work than usual.	1.59	0.67
SC_14	I have sleep problems [e.g., trouble falling asleep or staying asleep, or sleeping too much, or have nightmares].	2.00	0.50
	**Cognitive stress**	1.65	0.67
SC_7	I find it difficult to make decisions, and I check the issues in my mind over and over again, but they don’t become clear to me.	1.66	0.66
SC_10	I find it difficult to focus on my work.	1.64	0.66
SC_11	I find it hard to plan and think clearly.	1.65	0.67
SC_13	I misplace things I need to work, lose them or forget appointments or to do my due diligence.	1.65	0.69

**Table 2:** Summary of pre- and post-test stress scores.

Code	Place of Residence	Child Type	Pre-Test				Post-Test			
			C	E	P	Total	C	E	P	Total
628567	Ajman	Cognitive disability	10	15	13	38	7	10	12	29
617741	Ras Al Khaimah	Cognitive disability	9	16	14	39	7	10	13	30
621642	Ras Al Khaimah	Sensory disability	10	16	15	41	7	11	12	30
628583	Ajman	Cognitive disability	10	15	14	39	7	10	12	29
622416	Ajman	Cognitive disability	11	16	12	39	8	11	10	29
629560	Fujairah	Cognitive disability	9	17	15	41	6	11	14	31
624981	-	Developmental delay	11	18	9	38	6	12	10	28
616960	Umm Al Quwain	Cognitive disability	10	16	13	39	7	11	11	29
633223	Fujairah	Cognitive disability	9	16	15	40	6	10	13	29
627829	Ajman	Cognitive disability	9	15	13	37	7	12	11	30
632847	Fujairah	Cognitive disability	11	15	13	39	7	10	12	29
628653	Ajman	Cognitive disability	10	17	13	40	6	10	13	29

Note: C = cognitive stress; E = emotional stress; P = physical stress.

## Data Availability

The data that support the findings of this study are available from the corresponding author upon reasonable request.
